# The Role of Slow Gait Speed in Cognitive Decline among Aging Women: A Systematic Review

**DOI:** 10.24966/ggm-8662/100280

**Published:** 2026-02-03

**Authors:** Joel Acevedo-Nieto, Karen Martínez, Claudia Amaya

**Affiliations:** Medical Sciences Campus, University of Puerto Rico, San Juan, Puerto Rico

**Keywords:** Aging, Alzheimer’s Disease, Cognitive Decline

## Abstract

The prevalence of Alzheimer’s disease and related dementias are increasing at an alarming rate, with projections estimating that by 2060, approximately 13.8 million adults aged 65 years and older in the U.S. will be affected by one or both. Among the many symptoms associated with cognitive decline, gait impairment is one that significantly affects functional independence and mobility.

**Methods:**

A systematic review was conducted to analyze 49 peer-reviewed studies using the Covidence systematic review software and adhering to PRISMA guidelines. The selected articles examined variables related to gait speed, and cognition. Participants were assessed through validated neurocognitive and mobility measures, including the MoCA and Dynamic Gait Index.

**Results:**

A significant negative correlation was identified between usual walking speed and age. This trend was particularly pronounced in women, in whom a significant negative association between MoCA scores and age (P = −0.019) was observed, suggesting an increased susceptibility to cognitive deterioration with advancing age.

**Conclusion:**

These findings underline the sex-specific nature of the relationship between gait speed and cognitive function, highlighting increased vulnerability in aging women. The decline in mobility and cognition observed in this population underscores the urgency of developing targeted interventions that integrate physical and cognitive rehabilitation strategies.

## Introduction

Gait, a fundamental motor function, is intricately linked to brain health and cognitive performance. Walking not only enhances cerebral blood flow and oxygenation but also promotes the release of Brain-Derived Neurotrophic Factor (BDNF), a key modulator of neuroplasticity and synaptic remodeling [1–5]. These neurophysiological processes are essential for maintaining memory and executive functions such as attentional control—domains that commonly decline with age.

Recent research highlights reductions in gait speed as a potential early biomarker for cognitive decline in older adults [6,7]. However, mounting evidence suggests that this motor-cognitive link is sex-specific. Older women tend to exhibit slower gait speeds than older men and are more susceptible to age-related cognitive impairments, including Alzheimer’s disease and Alzheimer’s Disease–Related Dementias (ADRD). The prevalence of these conditions is significantly higher among women, who not only live longer than older men but also show steeper declines in executive function, memory, and motor performance [8]. This systematic review examines the association between Slow Gait Speed (SGS) and cognitive decline with a specific emphasis on sex-based disparities. By focusing on aging women—a group disproportionately affected by both physical frailty and neurodegeneration—we aim to underscore the need for sex-tailored interventions in aging research and clinical practice.

## Background

In the U.S., nearly half of individuals aged 60 years and older demonstrate SGS (<0.8 meters per second), with rates slightly decreasing from 48.6% (2006–2008) to 45.7% (2014–2016) [8]. However, epidemiological studies consistently report that women walk more slowly than men and experience greater declines in mobility with age [9].This slower gait is not merely a biomechanical issue—it has substantial implications for cognitive aging. Women are not only at higher risk for developing ADRD, but they also exhibit faster cognitive deterioration compared to men of similar age. Moreover, social determinants of health—such as lower lifetime physical activity levels and disparities in access to healthcare—further compound these risks in older women.

Understanding the role of gait speed in predicting cognitive decline, particularly in women, could inform early screening and prevention strategies. Identifying slow gait as a modifiable risk factor holds promise for the design of interventions that preserve both physical independence and cognitive integrity.

## Methods: Study Selection

Using the Covidence systematic review software, we analyzed 49 peer-reviewed studies following PRISMA (Preferred Reporting Items for Systematic Reviews and Meta-Analyses) guidelines. The studies (49 peer-reviewed) examined were analyzed gait limitations, physical activity, balance training and cognitive outcomes. The Cochrane framework was used to assess the risk of bias [10].

### Inclusion criteria

The systematic review included a range of study designs such as randomized controlled trials, cross-sectional studies, case-control studies and pilot studies. The target population consisted of adults aged 60 years and older, encompassing individuals who were cognitively healthy, those with mild cognitive impairment, and those diagnosed with Alzheimer’s Disease or Related Dementias (ADRD) [11,12]. The selected studies employed validated tools to assess gait performance, such as the Dynamic Gait Index, and cognitive function, including the Montreal Cognitive Assessment (MoCA) [13–15]. In addition, studies measuring Brain-Derived Neurotrophic Factor (BDNF) or related biomarkers were considered. Importantly, only studies that stratified outcomes by sex were included to enable a focused analysis of sex-specific differences [16].

### Exclusion criteria

Articles written in languages other than English or Spanish, as well as studies published before the year 2000, were excluded.

### Cognitive assessment

MoCA scores were classified into four categories: scores between 26 and 30 indicated normal cognition; scores from 18 to 25 reflected mild impairment; scores between 10 and 17 indicated moderate impairment; and scores below 10 were considered indicative of severe impairment.

### Data analysis

- Spearman’s rank correlation assessed gait-cognition associations by sex
- Trend analyses evaluated progressive changes in gait and cognition in older men and women
- P < 0.05 indicated statistical significance

## Results

### Sex-specific declines in gait and cognition

**Figure 1:**
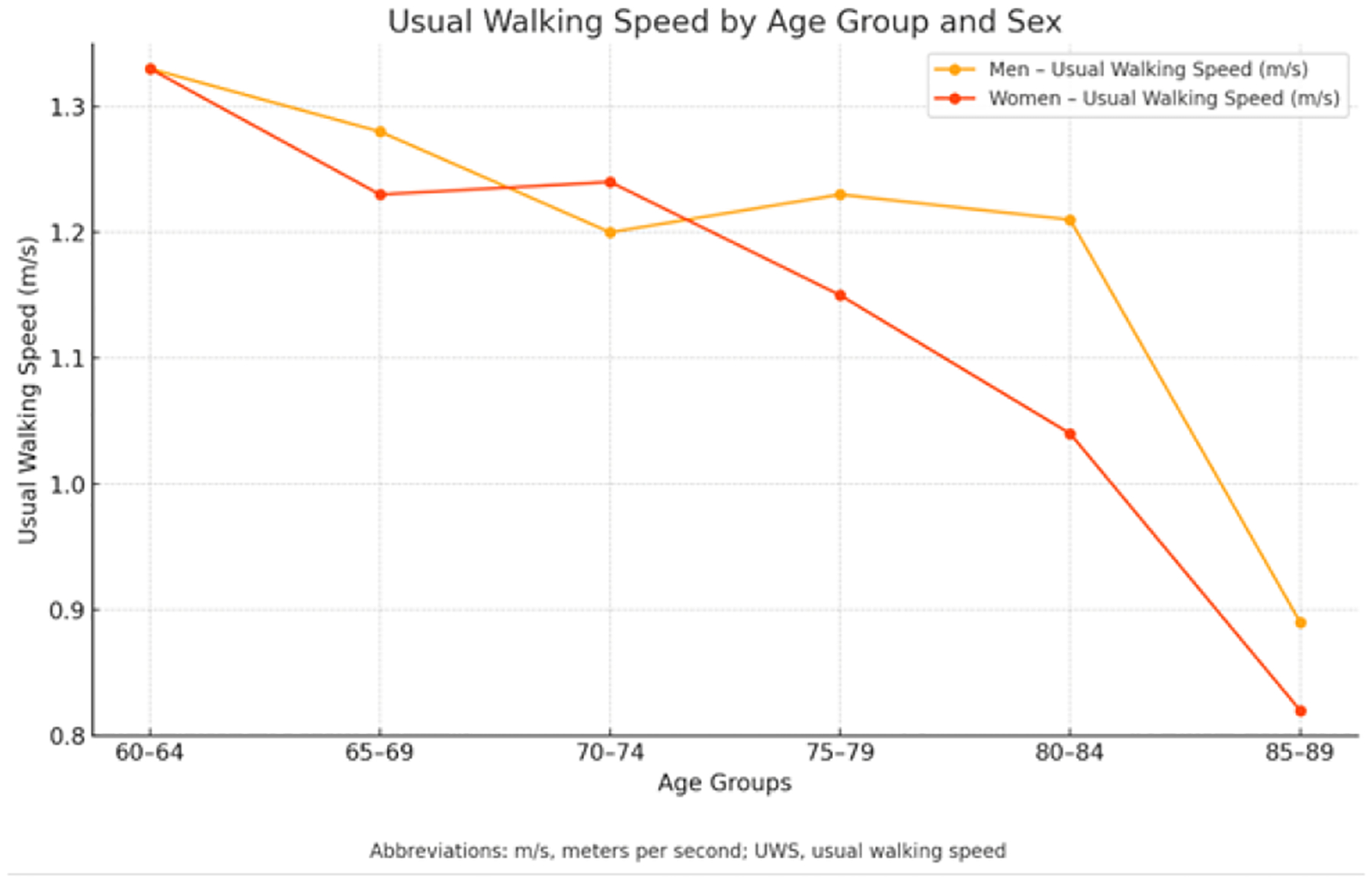
Usual Walking Speed by Age Group and Sex: This line graph illustrates the usual walking speed (in meters per second) across different age groups for men and women. Both sexes show a decline in walking speed with increasing age. Women show a sharper decline after age 75–79, with a notable drop in the 85–89 group. Men maintain a higher usual walking speed across all age groups.

**Figure 2:**
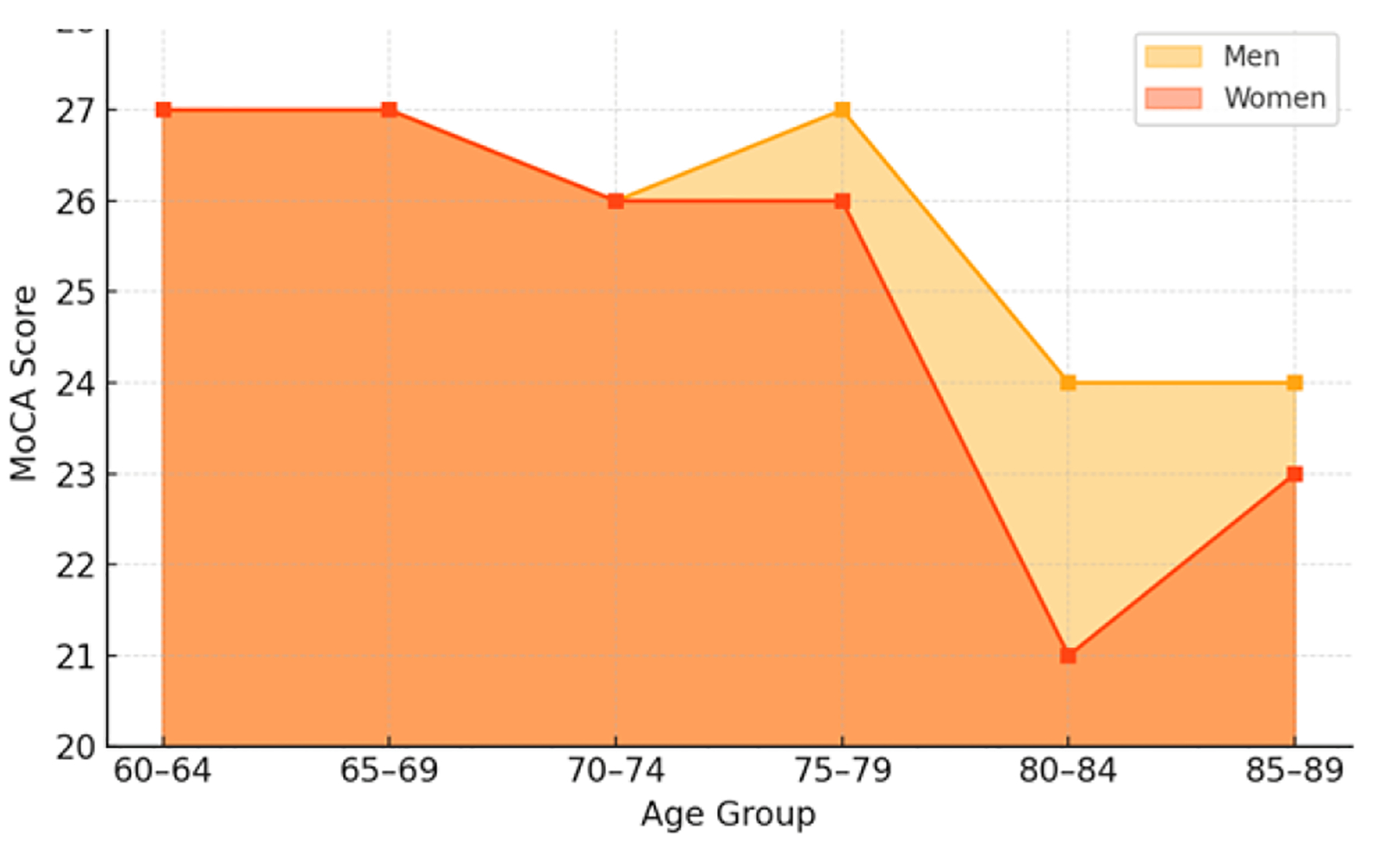
Montreal of Cognitive Assessment score by age groups and sex: This area graph shows MoCA scores across age groups for men and women. While scores remain relatively stable through age 70–74, a marked decline appears in both sexes beginning in the 75–79 group, with the most significant drop for women in the 80–84 group. Men demonstrate slightly higher cognitive performance overall across age groups.

Usual Walking Speed (UWS) decreased significantly with age in both sexes, r = 0.828 in men and r = 0.942 in women, (Figure 1) [17]. However, cognitive decline—as measured by MoCA—was significantly associated with age only in women, r = −0.019, P < 0.05, (Figure 2). In men, the relationship was weaker and non-significant (r = −0.0802, P = 0.064), suggesting greater cognitive resilience despite physical decline.

### Trend analysis

**Figure 3:**
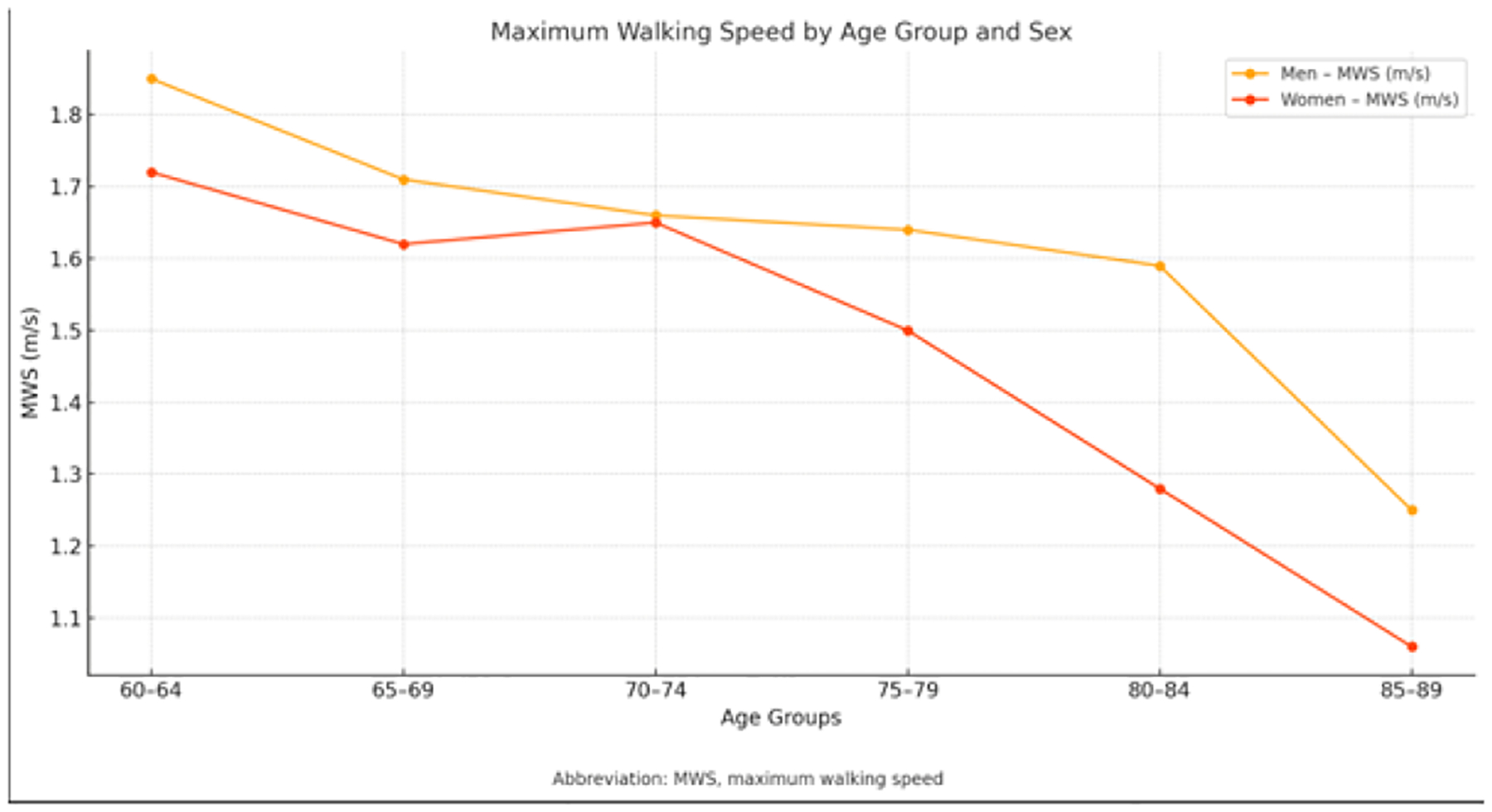
Maximum walking speed by age groups and sex: This graph displays maximum walking speed (in meters per second) by age group and sex. Men consistently exhibit higher maximum walking speeds than women. A steady decline is observed with age, with the largest decrease occurring in the 85–89 age group for both sexes.

- UWS declined more sharply in women (P = 0.01) than in men (P = 0.04)
- Maximum walking speed decreased significantly in both groups, with men (P = 0.004) showing a slightly steeper decline, though women, P = 0.014, (Figure 3), still exhibited significant loss
- Cognition: Only women showed a statistically significant decline in MoCA scores with aging (P = 0.032), reinforcing the hypothesis that gait, and cognitive decline are more tightly coupled in women

### Slow gait speed as a predictive marker in women

Women over age 80 showed the steepest drop in both gait speed and MoCA scores [18]. These dual declines suggest that SGS may serve as a non-invasive marker for early cognitive deterioration in women. Factors such as increased frailty, hormonal shifts, and accelerated muscle loss may contribute to women’s increased vulnerability to cognitive and physical decline.

## Discussion

### Shared neural pathways and sex-specific aging

Motor and cognitive processes share common neural substrates—in particular the prefrontal cortex, basal ganglia, and cerebellum. As women age, these structures may undergo accelerated degeneration, potentially explaining their heightened susceptibility to both gait and cognitive impairment.

### Clinical implications

By recognizing SGS as a red flag for cognitive decline—especially in women—clinicians can initiate earlier diagnostic and therapeutic interventions. Routine gait assessments are cost-effective and easy to implement in primary care and geriatric settings. By identifying at-risk individuals early clinicians can deploy preventive strategies that combine physical training, cognitive stimulation and nutritional interventions.

## Limitations and Future Research

This review is limited by challenges related to heterogeneity in study designs, gait measurement protocols and cognitive assessment tools. Longitudinal studies and more detailed domain-specific cognitive assessments are needed to clarify causal relationships. Furthermore, few of the studies examined incorporated neuroimaging or biomarkers (e.g., BDNF, inflammatory cytokines), either or both of which could elucidate underlying mechanisms.

## Conclusion

This systematic review provides further evidence of the strong, sex-specific relationship between SGS and cognitive decline. Women, in particular, show a more pronounced dual decline in mobility and cognition compared to men—likely due to a combination of biological vulnerability and sociocultural factors. Slow gait speed may be a valuable early biomarker for identifying cognitive risk in older women.

## Future Directions

- Longitudinal cohort studies with integrated gait, cognitive and neurobiological data
- Further investigation of hormonal and molecular drivers of aging in women
- Standardization of gait and cognitive assessments to enhance reproducibility
- Development of targeted interventions for mobility and cognitive resilience in aging women
